# Educating diabetic patients through an SMS intervention: a randomized controlled trial at a Brazilian public hospital

**DOI:** 10.20945/2359-3997000000390

**Published:** 2021-07-16

**Authors:** Viviane Moura Aceti, Rodolpho Vianna Santoro, Luis Guillermo Coca Velarde, Diego Nunes Brandão, Rubens Antunes da Cruz, Giselle F. Taboada

**Affiliations:** 1 Universidade Federal Fluminense Programa de Pós-Graduação em Ciências Médicas Niterói RJ Brasil Programa de Pós-Graduação em Ciências Médicas, Universidade Federal Fluminense, Niterói, RJ, Brasil.; 2 Centro Federal de Educação Tecnológica Celso Suckow da Fonseca Rio de Janeiro RJ Brasil Centro Federal de Educação Tecnológica Celso Suckow da Fonseca, Rio de Janeiro, RJ, Brasil.; 3 Universidade Federal Fluminense Instituto de Matemática e Estatística Niterói RJ Brasil Instituto de Matemática e Estatística, Universidade Federal Fluminense, Niterói, RJ, Brasil.; 4 Universidade Federal Fluminense Faculdade de Medicina Departamento de Medicina Clínica Niterói RJ Brasil Departamento de Medicina Clínica, Faculdade de Medicina, Universidade Federal Fluminense, Niterói, RJ, Brasil.

**Keywords:** Diabetes mellitus, health education, self-care, SMS

## Abstract

**Objective:**

Diabetes mellitus (DM) has a high healthcare system cost worldwide. Educational strategies are important to improve self-care and control this disease. This study aimed to evaluate satisfaction and clinical efficacy of a Short Message Service (SMS) educational intervention in self-care and nutrition at a Brazilian university hospital.

**Materials and methods:**

We conducted a trial of educational intervention and assigned eligible patients with DM to either receive weekly educational SMS for 6 months (intervention group [IG]) or no SMS at all (control group). A satisfaction questionnaire was applied before and after the intervention in both groups. Laboratory (fasting glucose, hemoglobin [Hb] A1c, total cholesterol, triglycerides, high-density lipoprotein, and low-density lipoprotein) and clinical (blood pressure) data were also collected. Data were analyzed using nonparametric tests with the Statistical Package for the Social Sciences.

**Results:**

We included 128 patients (64 in each group). Responses to the satisfaction questionnaire with self-care and healthcare professionals from 112 patients revealed an improvement in the perception of receiving information regarding helpful eating habits and in healthy eating behavior and an improvement in satisfaction with their diabetes care in the IG. In the post-intervention period, improved systolic blood pressure and HbA1c levels were observed in the IG as illustrated by delta % (post-intervention minus pre-intervention data divided by pre-intervention data multiplied by 100) reductions of 2.3% and 3.9%, respectively

**Conclusion:**

SMS intervention was useful as an educational tool for improving satisfaction and glycemic and blood pressure control of patients with DM observed at a Brazilian university hospital.

## INTRODUCTION

The World Health Organization (1) estimates the number of adults with diabetes mellitus (DM) to reach 629 million in 2045 with a more pronounced increase in low- and middle-income countries. In Latin America, a 55% increase in prevalence is expected (2). DM and other chronic noncommunicable diseases and their comorbidities have a high healthcare system cost worldwide. Global annual costs related to adults with DM were approximately US$ 850 billion in 2017 (1). In Brazil, direct and indirect costs of adult outpatients with DM treated by the Unified Health System (Brazilian Public Health System – *Sistema Único de Saúde*) were estimated at US$ 15.67 billion in 2014 (3). In that same year, at least an additional US$ 264.9 million was spent on hospitalizations for these patients (4). Thus, appropriate treatment and patient support are critical to prevent DM complications and consequently its economic and social burden (5,6).

Individuals with DM should be included in educational programs since diagnosis, with the main aims of raising awareness, training for self-care, and maintaining independence regarding lifestyle changes for adequate disease control (7). A remote educational methodology widely used today is mobile phone technology, which allows sending educational text messages to patients (5,8,9). Through “Short Message Service” (SMS), patients can receive information about their disease and guidelines for improving quality of life (5,8,10). Mobile telephony utilization in health education is promising because it is easy to implement since nowadays most people have a device capable of receiving messages and can minimally use its functionalities (11).

In Brazil, SMS for disease management is still underused, despite being successful in the dissemination of alerts by public agencies. SMS technology is a low-cost and wide-reaching alternative for the public health system that can be used for improving care in patients with chronic diseases (5,8,12,13). It is important to note that in many other countries, this approach has already been used with proven effectiveness in glycemic control (5,9–11,14–20), patient satisfaction (5,10,11,14,15,19), and cost reduction for healthcare systems (5,9,15,21), emphasizing the relevance of the present study. We created a batch SMS sending system requiring only the purchase of a data plan from any telecommunication company. Our study aimed to evaluate satisfaction and clinical efficacy of an educational intervention in self-care and nutrition at a Brazilian university hospital.

## MATERIALS AND METHODS

### Trial design

This study was a randomized controlled longitudinal clinical trial of an educational intervention based on a convenience sample.

### Patient population, study setting, and recruitment goals

We included 128 patients with DM followed up at a Brazilian public university hospital. The target population was chosen based on a previous study from our group (unpublished data), which found insufficient knowledge about DM, especially among older and undereducated patients. We also considered whether patients had and knew how to use a cell phone capable of receiving SMS text messages.

### Patient recruitment and randomization

This study was approved by the Research Ethics Committee of HUAP/UFF (CAAE 68463317.7.0000.5243) and registered at clinicaltrials.gov (NCT04191902 - 12/07/2019). The inclusion criteria were diagnosis of DM, aged older than 18 years, at least 1-year follow-up at the endocrinology clinic, and owning a cell phone. The exclusion criteria were amaurosis, illiteracy and pregnancy. All participants were presented to and signed the informed consent form before any study procedure. A brief interview was conducted to obtain sociodemographic data. Participants then answered the first part of the survey questionnaire relating to satisfaction with their self-care and medical care. The selection of the control group (CG) and the intervention group (IG) was performed in a random manner (1:1) using random block randomization. Information of each patient was stored in envelopes, which were subsequently sealed. Envelopes were shuffled and selected alternately for each group. The first envelope was for the IG, the second was for the CG, and so on, resulting in 64 patients in each group. Participants and staff were not blinded to group allocation. Clinical and metabolic control data from the last two visits (before study entry) were obtained from the patients’ medical records.

### Intervention

For six months, 64 participants in the IG received a weekly SMS, whose content was easy to read and understand, about self-care in DM, encouraging healthy eating and physical activity. Regarding self-care in DM, SMSs reminded patients to take their medication and/or insulin, instructed patients on how to check their blood pressure and glucose levels, and encouraged foot care. Regarding food, the messages addressed examples of suitable meals, foods to avoid, and fruits and vegetable suggestions according to the season. Regarding physical activity, messages informed which exercises patients could practice, for how long, and the importance of their regularity. We emphasized that all patients in both the IG and CG should continue to be regularly followed up at the endocrinology outpatient clinic, attending medical, nutrition, and nursing consultations as needed.

An SMS message sending system was developed for this study with two applications in Java language, allowing portability to several computer systems. The first application consists of a desktop version, whereas the second consists of an Android application. The system uses a wireless communication network to transfer data between the computer and the cell phone. The desktop version allows for loading of patients’ contact and specification of messages to be sent. The phone acts as a redirector; that is, the Android application developed receives a package containing all the information (messages and recipients) from the desktop application and, using the standard messenger on the smartphone itself, sends each message to the respective patient. Messages are identified with the name of patient, giving message a personal touch.

### Outcome measures

The primary outcome was to evaluate the influence of the SMS intervention on patient satisfaction with self-care. The secondary outcome was to evaluate this influence on clinical and laboratory parameters of DM control. Finally, feasibility of performing such an intervention was also evaluated.

### Satisfaction outcomes

To assess the impact of the intervention on patient satisfaction with their self-care and healthcare professionals and evaluate satisfaction with the SMS received, we used a questionnaire derived from a validated one (22). Our questionnaire included questions about patient satisfaction with their self-care and with healthcare professionals as presented in Table 1. All five questions had three answer options (always, sometimes, never), which scored 2, 1, or 0 points, respectively, resulting in a total score from 0 to 10. Moreover, the following questions were developed about satisfaction with the SMSs received: “Was the language used in the messages easy to understand?,” “Did you learn anything new from the messages?,” “Did the messages remind you to do something including check your blood sugar or eat healthy food?,” “Did you like receiving messages?,” and “Was the frequency of the messages adequate?” In the 60 days following the end of the intervention period, questions regarding satisfaction with self-care and with healthcare professionals were reapplied to both groups (control and intervention), whereas questions regarding satisfaction with SMS messages were applied only to the IG. To evaluate satisfaction with self-care and with healthcare professional outcomes, we compared each question score and total questionnaire score before and after the intervention using a new numeric variable delta (post- minus pre-intervention score).

**Table 1:** Satisfaction questionnaire with self-care and healthcare professionals: Brazil, 2019

		Control (n = 56)	Intervention (n = 56)	p value
“Do you receive information about helpful eating habits to improve diabetes control?”	Delta (Post – Pre)	0 (0-0)	0 (0-1)	**0.001** *
“Do you eat healthy?”	Delta (Post –Pre)	0 (0-0)	0 (0-1)	**0.001** *
“Do health professionals provide information that is easy to understand?”	Delta (Post – Pre)	0 (-1 - 0)	0 (0-0)	0.169
“Do you feel comfortable asking your doctors when you have questions?”	Delta (Post – Pre)	0 (0-0)	0 (0-0)	0.835
“Are you satisfied with your own diabetes care?”	Delta (Post – Pre)	0 (−1 - 0)	0 (0-0)	**0.002** *
Total (points)	Pre (total score)	7 (5-8)	6 (5-8)	0.868
Median (p25-p75)	Post (total score)	6 (4-7)	7 (6-8)	0.002
	Delta (Post – pre)	0 (−2 – 0)	1 (−1 – 2)	**<0.001** *

Data are presented as median (p25-p75). Total scores for satisfaction questionnaire before (pre) and after (post) the intervention as well as delta (post – pre) which corresponds to pos-intervention minus pre-intervention score. Mann-Whitney's test was used for comparison between groups.

* Statistically significant (P < .05) difference between the groups.

### Clinical and laboratory outcomes

To measure the influence of the intervention on patient health, we collected clinical and metabolic control data from the two consultations before and after the intervention period from patients’ medical records. Blood pressure data and the following laboratory parameters were evaluated, which were measured using standard available commercial kits: glucose, hemoglobin (Hb) A1c, total cholesterol, triglycerides, and high-density lipoprotein cholesterol (HDL-C). Low-density lipoprotein cholesterol (LDL-C) was calculated using the Friedewald formula (23).

### Feasibility outcomes

Feasibility results were evaluated from the number of patients who agreed to participate in the study, who met our inclusion and exclusion criteria, and who have reached the end of the present study. Besides, we developed an SMS sending system, which could be used by the public health system at a low cost while allowing for a significant number of patients to be reached remotely.

### Statistical analyses

The Kolmogorov-Smirnov test was performed to assess the normality of numerical variables, and all showed asymmetric distribution. Thus, numerical data are presented as median (p25-p75), and comparison between groups was made using the Mann-Whitney U test. Comparison of categorical variables between groups was made using Fischer's exact test. For the clinical and laboratory numerical variables (systolic and diastolic blood pressure, fasting glucose, HbA1c, total cholesterol, triglycerides, HDL-C, and LDL-C) we compared pre- and post-intervention data using percent variation (delta %), calculated as follows: post-intervention minus pre-intervention data divided by pre-intervention data multiplied by 100. This strategy was adopted to prevent effect size interference of the intervention (greater effect in poorly controlled patients). To evaluate satisfaction with self-care and with healthcare professional outcomes, we considered each question and total questionnaire score using a new variable (delta) calculated as follows: post-intervention score minus pre-intervention score, which was compared between the IG and CG, using the Mann-Whitney U test.

## RESULTS

### Feasibility outcomes

**Figure 1:**
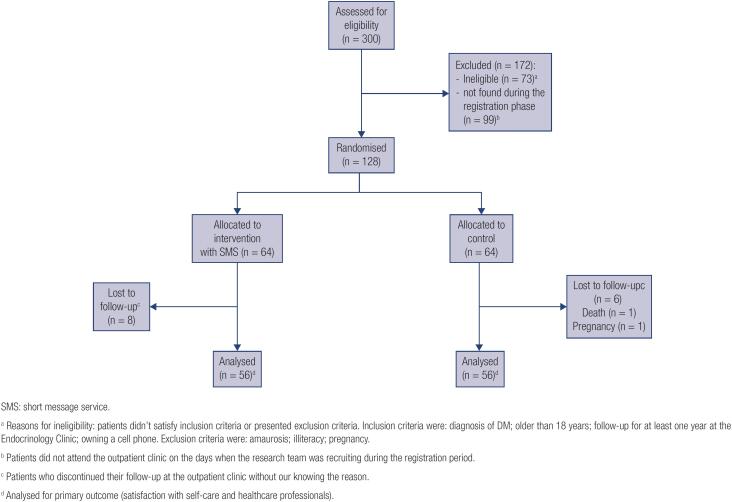
Patient sample selected: Brazil, 2018-2019

Regarding enrollment and follow-up, between January and December 2018, we assessed 300 patients for eligibility from a Brazilian public university hospital, where 128 patients were eligible and randomized (42.6%) (Figure 1). Among the ineligible patients, 73 did not meet the inclusion criteria or met the exclusion criteria, and 99 did not attend the outpatient clinic on the days when the research team was recruiting participants during the registration period. Primary outcome data were available for 112 patients (87% overall retention) after eight patients were lost to follow-up in each group. One patient died, another one became pregnant, and the others did not attend outpatient clinic appointments during the study period. Moreover, we were unable to contact these patients using the cell phone number provided when they were recruited. Reasons why they missed the appointments were not determined, and whether they returned for medical care continuation was not identified.

### Enrolled patient characteristics

One hundred twenty-eight (64 in the IG and 64 in the CG) patients were included. Most patients were women (60.9% vs. 68.8% in the IG and CG, respectively) with a median age of 42 years (39 vs. 45). Patients’ sociodemographic, clinical, and laboratory characteristics are shown in Table 2 and were similar, except for alcohol withdrawal, which was more frequent in the CG than in the IG (p = 0.012).

**Table 2:** Participants’ baseline demographic, clinical and laboratorial characteristics (n = 128): Brazil, 2018

Characteristic		Control (n = 64)	Intervention (n = 64)
Personal:			
	Sex (female)	44 (68.8)	39 (60.9)
	Age (years)	45 (29-61.8)	39 (27.25-58.8)
Social:			
	Elementary School	36 (56.3)	30 (46.9)
	Does not work	44 (68.7)	45 (70.3)
	Single/divorced/widowed	42 (65.7)	32 (50)
Lifestyle:			
	Non smokers	53 (82.8)	49 (76.6)
	No-alcohol	51 (79.7)	38 (59.4)
Medical history:			
	Type 1 diabetes	33 (54.1)	36 (56.3)
	Diabetes years	15 (8.5-22)	15 (9-20)
	Oral antidiabetic only	9 (14.8)	8 (12.5)
	Oral antidiabetic + insulin	25 (41)	28 (43.8)
	Insulin only	27 (44.3)	28 (43.8)
Clinical:			
	SBP	123 (113.5-140)	125 (115-145)
	DBP	80 (70-85)	80 (75-85)
	FG	154.3 (107.6-198)	163.8 (112.4-223.4)
	HbA1c	8.0 (7.1-9.8)	9.7 (7.7-10.8)
	TC	170 (143-205.5)	183.5 (159.5-207.5)
	Tg	96.5 (61.5-147.8)	94 (67-144.3)
	HDL	46.25 (39.1-67.5)	50.8 (42.3-68.5)
	LDL	94 (71.8-123.5)	102.5 (82-123.8)

Data are presented as Median (p25-p75) or n of Patients (%).

SBP: systolic blood pressure; DBP: diastolic blood pressure; FG: fasting glucose; TC: total cholesterol; Tg: triglycerides; HDL: high-density lipoprotein; LDL: low-density lipoprotein.

### Satisfaction outcomes

Responses to the satisfaction questionnaire with self-care and healthcare professionals from 112 patients revealed an improvement in the perception of receiving information regarding helpful eating habits (p = 0.001) and in healthy eating behavior (p = 0.001) and an improvement in satisfaction with their diabetes care (p = 0.002) in the IG (Table 1).

Among the 56 patients from the IG who answered the SMS satisfaction questionnaire, no “never” answer was obtained. All patients enjoyed receiving messages and considered them easy to understand. Moreover, 75% and 76.8% of the patients answered “always” to the questions “Did you learn anything new from the messages?” and “Did the messages remind you to do something including check your blood glucose or eat healthy food?,” respectively. Furthermore, 82.1% of the patients answered “always” to the question “Was the frequency of the messages adequate?”

### Laboratory outcomes

In the post-intervention period, improved systolic blood pressure (p = 0.017) and HbA1c levels (p = 0.008) were observed in the IG as illustrated by delta % (post-intervention minus pre-intervention data divided by pre-intervention data multiplied by 100) reductions of 2.3% and of 3.9%, respectively. There was no statistically significant difference in any other clinical or laboratory parameters between groups (Table 3).

**Table 3:** Participants’ clinical and laboratorial characteristics before and after intervention: Brazil, 2019

		Control	n_o_	Intervention	n_i_	p value
SystBP	Pre	125 (115-140)	59	125 (115.8-145)	56	
	Post	125 (115-144)		120.5 (115-135)		
	Delta (%)	3.5 (−7.7-10.8)		−2.3 (−10.6-4.1)		**0.017** *
DiastBP	Pre	76 (70-85)	59	80 (75-86.9)	56	
	Post	75 (65-80)		75 (70-82.5)		
	Delta (%)	-2.8 (-11.1-7.7)		-5.6 (-11.9-6.0)		0.571
FG	Pre	150.5 (107.5-197)	55	162.5 (103-202)	58	
	Post	151.5 (96.4-204.1)		144.5 (111-196)		
	Delta (%)	2.6 (-33,1-50.8)		-7.8 (-23.7-26.2)		0.861
HbA1c	Pre	8.0 (7.1-9.8)	58	9.7 (7.7-10.9)	58	
	Post	8.5 (7.7-10.1)		8.4 (7.2-10.3)		
	Delta (%)	2.1 (-4.1-11.8)		-3.9 (-12.4-4.6)		**0.008** *
TC	Pre	171 (143.3-209)	52	180.5 (157-204)	55	
	Post	178 (141.8-200.5)		184 (154.8-204.5)		
	Delta (%)	0.9 (-9.2-11.3)		-0.8 (-9.5-7.1)		0.489
Tg	Pre	102 (61,5-147.8)	52	93.5 (67-124)	53	
	Post	99.5 (59.3-157.8)		87 (51-158)		
	Delta (%)	0 (-21.1-21,6)		-10.5 (-29.9-30.3)		0.303
	Pre	46.3 (39.6-57.5)	33	50 (41.5-69)	44	
	Post	47 (39.5-1.5)		54 (40.8-70.3)		
HDL	Delta (%)	3.8 (-6.4-11,0)		5.2 (-4.9-11.8)		0.853
LDL	Pre	94 (73-125.5)	41	102.5 (80.8-123.8)	46	
	Post	93 (76-123.5)		109 (81.5-137)		
	Delta (%)	0 (-19.5-17.2		5.2 (-15.2-18.0)		0.638

Delta: (post-pre)*100/pre; SystBP: systolic blood pressure; DiastBP: diastolic blood pressure; FG: fasting glucose; TC: total cholesterol; Tg: triglycerides; HDL: high-density lipoprotein; LDL: low-density lipoprotein; nc: number of patients analysed in the control group; ni: number of patients analysed in the intervention group; Mann-Whitney's test was used for comparison between groups.

* Statistically significant (P < .05) difference between the groups.

### Qualitative analysis

During the study, while receiving SMSs, approximately 10% of the patients sent back several messages of gratitude. During the post-intervention period, while answering study questionnaires, all patients from the IG reported feeling motivated by the messages to take better care of their health. Approximately 20% of the patients requested to continue receiving messages or wanted to indicate acquaintances with DM.

## DISCUSSION

The present study evaluated satisfaction, clinical efficacy, and feasibility of an educational intervention in patients with DM using text messages via cell phone “SMS” and found promising results. It is worth mentioning that, until the submission date, no Brazilian study was found using this type of intervention for patients with DM treated in the public health network. Different international studies show that SMS use for health education of patients with DM has resulted in an improvement not only in behavioral habits but also in biochemical parameters of DM control (11,14–18,24,25). Another advantage to be highlighted is that health education approach through SMS has greater acceptance among patients than traditional approaches, which require reading pamphlets or attending lectures (14). Moreover, SMS does not require patient to own a smartphone or Internet, besides being easy to access and read at any time and place (15).

The study sample had significant representative characteristics of patients followed up at our hospital (older patients with long disease duration and multiple microvascular complications and comorbidities). This way, using convenience sampling probably did not introduce a bias in relation to patients with DM from a tertiary care unit. Thus, despite the limitations of non-probabilistic sampling, we believe our exploratory study yielded encouraging results that have excellent potential to be replicated with a larger number of participants.

The operational and economic viability of the study was made possible through the development of a batch SMS sending system. Our system presented limitations regarding SMS preparation since messages had to be typed and could not be saved to be used again later. A new version could have a message database, allowing the user to select the desired message to be sent. Concerning economic viability, currently, most telecommunication companies operating in Brazil have low-cost plans that include unlimited free SMS. In contrary, the Brazilian Government already uses a free SMS sending system for disaster alerts, which could also be used to send health educational messages.

DM prevalence has been reported to range from 7.2 to 27.6% in persons aged 35 to 44 and 55 to 64 years, respectively (26). This cohort study included patients aged 35 to 74 years and found a higher prevalence of DM in elderly, obese, and undereducated individuals. Costs increase with longer disease duration related to the development of chronic complications and can reach a 23% increase in those with DM for 20 years or more (27). In the present study, we were able to reach a group of patients reasonably representative of the Brazilian DM population with a median age of 42 years and particularly those with long disease duration (median, 15 years) and undereducated (approximately half studied up to elementary school) who are expected to produce higher costs for the public health system.

Satisfaction with self-care and healthcare professionals was assessed through a questionnaire that was derived from a validated tool (22) to improve comprehension and facilitate completion by participants. SMS intervention had no impact on dimensions that refer to the personal contact with the healthcare professionals (“Do healthcare professionals provide information that is easy to understand?” and “Do you feel comfortable asking your doctors when you have questions?”). This could have been anticipated, for, in this regard, sympathetic and empathetic factors can have a significant effect. This is even more challenging when one considers that the medical team changes periodically since our facility is a university hospital with training doctors. An improvement was observed both in the perception of receiving guidance on proper nutrition and in healthy eating habits. Although this evaluation was rudimentary, since the use of a 24-hour recall or a 3-day food record is complicated and was outside the scope of this study, this improvement may be an indicative of the adequacy of the SMS content. A nutritionist wrote messages with simple tips on healthy eating and seasonal fruits and vegetables for grocery purchase planning. Besides, participants were encouraged to seek assistance from the nutrition service for regular follow-up as part of the multidisciplinary approach to DM control. Finally, we found greater satisfaction with self-care in DM among patients in the IG. By promoting self-management support and self-sufficiency (19), core elements for optimizing patients with chronic diseases, we were able to achieve one of the main objectives of our messages.

All survey participants enjoyed receiving SMS, which confirms satisfaction with the intervention. This result was above our expectations and may be biased by participants not willing to provide a negative review of the study intervention. To minimize this problem, the research team was particularly thorough in explaining to participants that a sincere and reliable response was welcomed, and that “negative” reviews could be useful for adjustments in the type and format of future interventions. Qualitative results of this study support reliability in participant's positive reviews and are supported by reports of motivation to take better care of their health. As mentioned, participants sent messages expressing gratitude for receiving SMS and their interest in continuing to receive messages after the end of the study (data not collected objectively but manifested by many patients via SMS or in-person). This phenomenon has also been observed in another study (17) and seems consistent, reaffirming patient satisfaction in receiving messages.

The comprehension of message content was essential to assess the effectiveness of the intervention. In a review study (20), the authors found that patients enjoyed having a source of information to help them deal with their diet and lifestyle changes. Additionally, the authors suggest that the best SMS intervention model for managing DM should involve weekly messages for a short period (3 months). In another study (21), the authors believe that to maintain patients’ interest for an extended period, SMS content should be followed up by telephone calls for personal contact with the healthcare team. Given these considerations, we conclude that the intervention used in the present study with weekly SMS of varied and personalized content, for 6 months, was a satisfactory intervention that could be successfully reproduced in other populations.

A limitation to the applicability of our study is functional illiteracy that, according to statistics from the Brazilian Institute of Geography and Statistics (2018), affects approximately 38 million people in Brazil (28). This leads to a second limitation of our study, which is the satisfaction questionnaire we used that has not been validated. In a preliminary phase of the study, the original validated questionnaire (22) was provided to patients. However, since overall educational level was low (approximately half of the patients studied up to elementary school), we identified the need to adapt questions and limit the number of answering options to improve comprehension. We used a simple score metric to evaluate each questionnaire answer, and a total questionnaire score in which higher scores imply higher satisfaction and positive deltas (post minus pre) implies improved satisfaction. Moreover, we used a nonparametric (Mann-Whitney U test) test, which compares positions and not the questionnaire score itself, minimizing the problem of not using a validated tool.

Regarding the clinical and laboratory parameters, after 6 months of receiving weekly SMS, the IG had a 2.3% reduction in systolic pressure and a 3.9% reduction in HbA1c, whereas the CG showed 3.5% and 2.1%, respectively, increase in these parameters. These are satisfactory results for a non-pharmacological intervention. Even modest reductions in blood pressure may play a role in reducing microvascular complications and major cardiovascular events in patients with DM (19). We found only one study of SMS educational intervention in DM in which blood pressure parameters were evaluated (29). The 6-month daily SMS intervention also resulted in high satisfaction and acceptability ratings and HbA1c reduction such as our study. However, no statistically significant effects were observed for secondary clinical indicators such as blood pressure levels. It is worth noting that intervention in that study and in ours lasted 6 months and that interventions of longer duration may result in loss of beneficial effect on HbA1c, requiring adaptations to maintain efficacy, as previously discussed. In fact, in a recent meta-analysis (21), SMS educational intervention was shown to be effective in improving glycemic control in the first 6 months. The reduction in HbA1c observed in our study as a result of a 6-month weekly educational SMS intervention confirms reports of several original studies and meta-analyses in different populations and countries with varying levels of development. Starting from the initial median HbA1c level in our IG, the use of SMS has contributed to a 0.38% reduction in this parameter. This is in accordance with the previously reported reductions of 0.38–0.8% in HbA1c values with the use of SMS strategy in the management of patients with DM (15,16,18,29,30), which is significantly encouraging, considering the low cost and potential coverage of the intervention.

It is important to note that although not statistically significant, a clinically relevant difference was observed in baseline HbA1c levels between groups, with higher levels in the IG than in the CG. This could have favored the benefit from the intervention, through effect size interference, in which a greater effect is observed in poorly controlled individuals. However, a strategy was adopted to solve this problem, which was to compare not only HbA1c but also all numerical pre- and post-intervention variables using a percent variation (delta %). For this reason, we strongly believe that the clinical benefit in improved glycemic control has resulted from the study intervention.

Finally, a low-cost intervention with weekly educational SMS to patients with DM of long disease duration and low educational level was viable and effective, with high satisfaction among patients and resulting in a reduction in HbA1c levels. Future studies with long-term duration for patients with chronic diseases are considered necessary, emphasizing that, to keep patient interest, it is necessary to improve the messages within each topic addressed.
